# Regulation of Suppressors of Cytokine Signaling as a Therapeutic Approach in Autoimmune Diseases, with an Emphasis on Multiple Sclerosis

**DOI:** 10.1155/2011/635721

**Published:** 2011-11-01

**Authors:** Vinod S. Ramgolam, Silva Markovic-Plese

**Affiliations:** ^1^Department of Cardiology, Yale Cardiovascular Research Center, Yale School of Medicine, New Haven, CT 16511, USA; ^2^Department of Neurology, University of North Carolina at Chapel Hill, Chapel Hill, NC 27599, USA; ^3^Department of Microbiology and Immunology, University of North Carolina at Chapel Hill, Chapel Hill, NC 27599, USA

## Abstract

Multiple sclerosis (MS) is an inflammatory demyelinating, presumably autoimmune disease of the central nervous system (CNS). Among the available MS therapies, interferon (IFN)**β** and the recently introduced statins have been reported to exert their immunomodulatory effects through the induction of SOCS1 and SOCS3 in various inflammatory cell subsets. The SOCS proteins negatively regulate cytokine and Toll-like receptors- (TLR-) induced signaling in the inflammatory cells. SOCS1 and SOCS3 have been reported to play an important role in the regulation of Th17-cell differentiation through their effects on the cells of the innate and adaptive immune systems. IFN**β** and statins inhibit Th17-cell differentiation *directly* and *indirectly* via induction of SOCS1 and SOCS3 expression in monocytes, dendritic cells (DCs), and B-cells. Due to their rapid induction and degradation, and SOCS-mediated regulation of multiple cytokine-signaling pathways, they represent an attractive therapeutic target in the autoimmune diseases, and particularly relapsing remitting (RR) MS.

## 1. Introduction

MS is a chronic progressive CNS inflammatory disease, in which the autoimmune response against the CNS myelin proteins leads to a chronic inflammatory response [1, 2]. During the inflammatory response, autoreactive T-cells migrate through the otherwise impermeable blood-brain barrier into the CNS perivascular areas, where they cause demyelination and axonal degeneration. The chronic inflammatory response leads to neuronal conduction deficits associated with neurological symptoms and eventually results in the loss of functional neuronal tissue. Several immunomodulatory and immunosuppressive therapies (IFN*β*, glatiramer acetate, fingolimod, natalizumab, and mitoxantrone) are used in clinical practice as effective therapies in controlling disease activity and disability progression in patients with RR MS. 

 Th17-cells have been shown to play a critical role in the development of autoimmune responses in several autoimmune diseases, including MS, rheumatoid arthritis, psoriasis, juvenile diabetes, ulcerative colitis, and autoimmune uveitis [3, 4]. Th17-cells are identified in the CNS MS lesions, cerebrospinal fluid, and among the blood-derived mononuclear cells from MS patients, implying that they may play a critical role in the immunopathogenesis of MS [5–8]. Our laboratory has identified increased numbers of Th17-cells and an increase in the Th17-cells' master regulatory transcription factor, retinoic acid-related orphan nuclear factor (RORc), and cytokines that mediate Th17-cell expansion [8] in MS lesions. Th17-cell frequency is approximately 7 times higher in the peripheral circulation of RR MS patients in comparison to healthy controls (HCs), and it positively correlates with the clinical disease activity [9]. While multiple studies have reported on the association of Th17-cells and autoimmune diseases, only several reports have provided a mechanistic data on the function of IL-17A in the development of the autoimmune response. IL-17A induces the secretion of CXCL1 and CXCL2, neutrophil-attracting cytokines, which attract those inflammatory cells into the CNS in the early or acute phase of the disease [10]. IL-17A together with IL-22 induces blood brain barrier permeability due to their effect on the endothelial cells [11]. IL-17A induces production of additional proinflammatory cytokines, including IL-1 and IL-6 [12], and cytokines coexpressed by Th17-cells, IL-9, IL-21, IL-22, and GM-CSF, also contribute to the development of the autoimmune response. 

SOCS proteins are a family of intracellular cytokine-inducible proteins, consisting of 8 members (CIS and SOCS1-SOCS7). They have two structural components: an Src homology domain (SH2), which is involved in phosphotyrosine binding to activated signaling molecules and a C-terminal SOCS box involved in the degradation of signaling molecules through the ubiquitin-proteasome pathway [13]. SOCS gene expression is triggered by interleukins, interferons, haematopoietic growth factors, and TLR ligands such as lipopolysaccharides (LPS) and CPG-containing DNA [13, 14]. The induction of the SOCS proteins occurs through cytokine-mediated activation of the Janus kinase/signal transducers and activators of the transcription (JAK/STAT) signaling pathway, which leads to the phosphorylation of the STAT transcription factors. The SOCS proteins negatively regulate cytokine signaling through their association with phosphorylated tyrosine residues on JAK proteins and/or cytokine receptors or by inhibition of STAT binding to the cytoplasmic domain of the receptors. They terminate the inflammatory responses by mediating proteasomal degradation of the bound proteins [13, 15, 16]. Among the SOCS family members, SOCS1 and SOCS3 have been described to play the most important role in the regulation of the autoimmune response. They are rapidly induced and degraded. They block phosphorylation-dependent activation of STAT1 in response to IFN-*γ*, or STAT3 in response to IL-6, and target the IFN*γ*R- and IL-6R-signaling complexes for proteasomal degradation [17]. Interestingly, two recent large genetic studies have identified SOCS1 as one of the genes with the strongest association with the susceptibility for MS [18, 19].

Among the available MS therapies, IFN*β* and the recently introduced statins have been reported to exert their immunomodulatory effects through the induction of SOCS1 and SOCS3 in various inflammatory cell subsets. IFN*β* and statins inhibit Th17-cell differentiation *directly* and *indirectly* via their effects on antigen-presenting cells (APCs). In this paper, we will discuss their therapeutic effects in RR MS patients, which are indirectly mediated through the induction of SOCS1 and SOCS3 in monocytes, DCs, and B-cells as well as directly affecting the Th17-cells.

## 2. Th17-Cell Differentiation

Similar to Th1- and Th2-cells, the differentiation of Th17-cells is orchestrated by cytokines secreted by APCs. The differentiation of mouse and human Th1-cells occurs upon exposure to IL-12 and IFN-*γ* and Th2-cells upon exposure to IL-4 and IL-10, whereas the differentiation of mouse and human Th17-cells differ in the required Th17-polarizing cytokines [20, 21]. TGF-*β* and IL-6 are required in the mouse, while IL-6, IL-1*β*, and IL-23 drive human Th17-cell differentiation [22–24]. Specific STAT molecules are involved in the differentiation of each T-cell subset. Th1-cells' differentiation induced by IFN-*γ* and IL-12 mediate activation of STAT4 and STAT1, which directly control the transcription factor T-bet. In the differentiation of Th2-cells, IL-4-induced STAT6 phosphorylation is crucial for the gene transcription of the master regulatory transcription factor GATA-3 [25], whereas STAT3 is pivotal in human and mouse Th17-cell differentiation, where it is induced by IL-6, IL-23, and IL-21 [26]. These studies have demonstrated a pivotal role of STAT molecules in T-cell differentiation. STATs' regulation is critical for the cytokine secretion profile and the subsequent inflammatory T-cell responses.

## 3. The Role of SOCS1 in the Inflammatory Response

SOCS1 is induced by various cytokines, including IFN*β*, IFN*γ*, IL-4, and IL-6 [27, 28], which activate the Jak/STAT signaling pathway. Several studies have demonstrated that SOCS1 is induced by the TLR4 (LPS) and TLR9 ligands (CPG-DNA) [14, 29]. SOCS1 classically inhibits IFN signaling through association with the IFN-*α* receptor 1 (IFNAR1) and IFN-*γ* receptor (IFNGR) subunits and suppression of IFN-induced STAT1 and STAT3 phosphorylation [30–32]. In addition to IFN regulation, SOCS1 also inhibits TNF-*α* signaling [14, 29]. SOCS1 deficiency leads to overresponsiveness to IFN-*γ*, whereas SOCS1 overexpression leads to a reduced responsiveness to IFN-*γ* in various cell subsets [31, 33, 34], implying that SOCS1 plays a negative regulatory role in IFN-*γ* signaling.

## 4. SOCS3 Regulates Cytokine Signaling

SOCS3 is expressed in DCs, monocytes, T-cells and B-cells upon induction by IL-2, IL-3, IL-6, IL-12, IL-23, IFN*α*/*β*/*γ*, IL-27, IL-4, IL-10, IL-1, TGF*β*, TNF-*α*, GM-CSF, and LPS. SOCS3 expression is high in resting CD4 cells, but it rapidly decreases after T-cell receptor activation. Earlier studies have reported that during T-cell differentiation, SOCS3 is selectively expressed in Th2-cells, while SOCS1 expression is higher in Th1-cells. High SOCS3 expression in transgenic mice led to skewing to Th2 type differentiation, because SOCS3 binds to IL-12RB2 and inhibits the IL-12-mediated STAT4 activation, therefore blocking Th1-cell development [35]. SOCS3 inhibits IL-6 signaling by binding to the IL-6 gp130 receptor complex and mediating its proteasomal degradation [36, 37]. The expression of SOCS3 negatively regulates STAT1 and STAT3 phosphorylation. During Th17-cell differentiation, STAT3 induces the expression of the master regulatory transcription factor RORc and is, therefore, critical for the differentiation of human and mouse Th17-cells [26, 38, 39]. The suppression of STAT3 phosphorylation is an effective mechanism for suppressing Th17-cell differentiation. A SOCS3 deficiency in naïve T-cells leads to a sustained STAT3 phosphorylation and results in higher frequencies of differentiated Th17-cells [40]. In addition, SOCS3 deficiency in T-cells is associated with enhanced IL-17A production, induced by IL-23 or IL-6 plus TGF-*β* [40]. IL-27 signaling can also induce SOCS3 via STAT1 so that IL-27 may block Th17-cell development, as may type I and II IFNs, via sequential activation of STAT1 and SOCS3, resulting in STAT3 antagonism [41]. Monocytes, CD4^+^ and CD8^+^ T-cells were found to have a lower SOCS3 and increased STAT3 expression during MS relapses [42]. Furthermore, SOCS3 suppresses the Th17-polarizing cytokine IL-1*β* and IL-23 secretion by DCs and B-cells [43, 44]. SOCS3 is induced by several stimuli in APCs, where it plays an important role in the inhibition of Th17 polarizing cytokines IL-1*β* and IL-23, which suppress STAT3 activation required for the gene transcription of the master Th17 cell regulatory transcription factor RORc. These studies suggest that SOCS3 induction may represent a beneficial therapeutic approach in patients with RR MS.

## 5. The Role of IFN*β*-Induced SOCS Expression in the Treatment of RR MS

IFN*β* is an innate immune response cytokine that suppresses the disease activity and disability progression of RR MS and its animal model, experimental autoimmune encephalomyelitis (EAE). Earlier studies in mice and humans have demonstrated an association between an endogenous IFN*β* deficiency and an increased susceptibility for EAE and RR MS [45, 46]. In addition, the administration of exogenous IFN*β* effectively reduced the clinical relapse rate and the formation of new CNS lesions in several large placebo-controlled clinical trials [47]. An increase in the Th1 receptor IL-12R*β*2 expression and the secretion of the immunoregulatory cytokine IL-10 [48, 49] were identified as the biomarkers of IFN*β*'s therapeutic effects. Multiple proposed mechanisms of IFN*β*'s therapeutic effect include inhibition of antigen presentation, suppression of T-cell proliferation and migration, and modulation of proinflammatory cytokine production [48, 49]. More recent studies have shown that IFN*β* effectively suppresses Th17-cells differentiation in mouse and human. Mice deficient for IFNAR1 receptor and its downstream signaling molecules have been found to be more susceptible to EAE [50, 51]. In addition, Durelli et al. have demonstrated that IFN*β* reduced the numbers of Th17-cells in their longitudinal study of IFN*β*-1a-treated RR MS patients [9]. This finding has been supported by our *in vitro *experiments on the effect of IFN*β* on human Th17-cell differentiation, where we demonstrated that IFN*β* suppresses Th17-cell differentiation via its effects on monocytes, DCs, B-cells and naïve T-cells [43, 44, 52]. 

**Figure 1:**
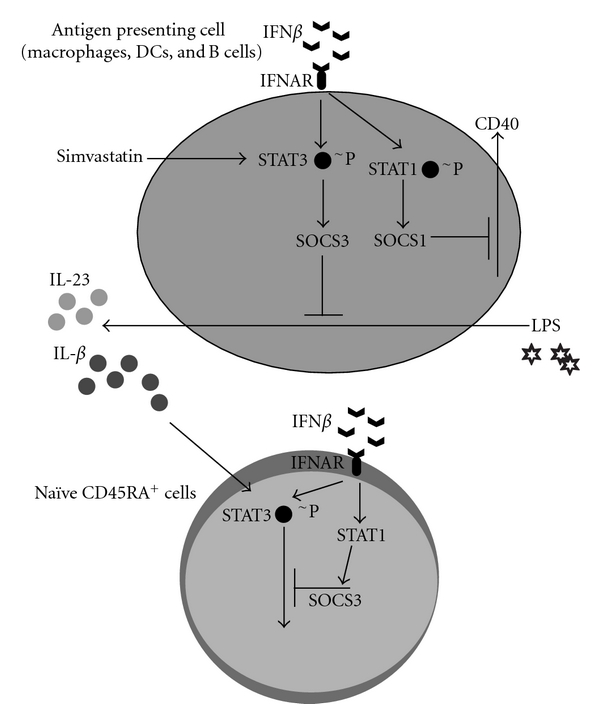
IFN*β* and simvastatin induce expression of SOCS1 and SOCS3. IFN*β* induces the phosphorylation of STAT1 and STAT3. STAT3 phosphorylation leads to the induction of SOCS3 expression, which downregulates the expression of IL-23 and IL-1*β*. The cytokines IL-23 and IL-1*β* promote the development of Th17-cells. In B-cells, the induction of STAT1 results in the expression of SOCS1, which downregulates the costimulatory molecule CD40. IFN*β* also acts on naïve CD45RA by inducing STAT1 phosphorylation, which subsequently induces SOCS3 that suppresses the STAT3 activation responsible for RORc gene transcription. Simvastatin induces the phosphorylation of STAT3 in APCs that leads to the expression of SOCS3, which in turn inhibits the gene transcription of IL-1*β* and IL-23.

Monocytes, macrophages, DCs and B-cells induce human Th17-cell differentiation through their secretion of IL-1*β* and IL-23. Li et al. have reported an increased expression of IL-23p19 in acute MS brain lesions, while Vaknin-Dembinsky et al. identified an increased synthesis of IL-23 by DCs derived from MS patients [53, 54]. In our *in vitro* experiments, we found a decreased expression of IL-1*β* and IL-23, whereas IL-12p35 and IL-27 gene expression was increased in IFN*β*-treated DCs [54]. RR MS patients treated with IFN*β* have been reported to have reduced IL-23p19 gene expression in their peripheral blood mononuclear cells (PBMCs) [49], a finding that is reinforced by our *in vitro* experiments showing that DCs and B-cells exhibited decreased IL-23 secretion upon incubation with IFN*β* [43, 44, 52]. We also demonstrated that the reduced secretion of IL-1*β* and IL-23 in supernatants (SNs) from IFN*β*-treated DCs led to a decreased differentiation of Th17-cells. The addition of IL-1*β* and IL-23 and the blocking of IL-27 in the SNs from IFN*β*-treated DCs and B-cells lead to a reversal of the IFN*β* effect and an increase in Th17-cell differentiation [43, 44]. In* IFNAR* knock-out mice that are highly susceptible to EAE, the EAE is reversed with IL-27 administration [51]. These studies led us to conclude that IFN*β* suppresses the Th17-cell differentiation by inhibiting IL-1*β* and IL-23 and inducing IL-27 secretion in DCs and B-cells as shown in Figure 1.

IFN*β* is a potent inducer of SOCS1 and SOCS3, molecules that contribute to Th17-cell differentiation [43, 55, 56]. We observed an increased expression of SOCS3 in IFN*β*-treated DCs, which is induced through STAT3 activation [43, 44]. Several studies have reported that SOCS3 suppresses IL-1*β* and IL-23 expression [57, 58]. Collectively, these reported *in vitro* and *in vivo* findings indicate that IFN*β* suppresses the IL-1*β* and IL-23 expression through SOCS3 upregulation in DCs and B-cells [43, 44]. 

We found that B-cells from RR MS patients and HCs exhibit an increased SOCS1 expression upon IFN*β*-induced STAT1 phosphorylation [44]. *In vivo *studies by Liu et al. have demonstrated that IFN*β*-1b treatment of RR MS patients inhibits the CD40 co-stimulatory molecule expression on B-cells [59]. We further identified in our experiments that IFN*β* inhibited CD40 expression on B-cells through the induction of SOCS1 [44]. STAT1 phosporylation is required for SOCS1 expression, which negatively regulates CD40 expression. STAT1 inhibition with fludarabine leads to increased CD40 expression in B-cells and the reversal of IFN*β*-1b's *in-vitro* effect [44, 55]. Similar findings have been reported for the IFN-*γ*- and IFN-*β*-induced CD40 expression on macrophages [60]. 

In a recent study, we have demonstrated that SOCS1 plays an important role in the regulation of B-cell CD40 expression and subsequently on their antigen presenting capacity [44]. The T-cell proliferation decreased when IFN*β*-pretreated B-cells were used as APCs and cocultured with the antigen-specific T-cells. In contrast, the proliferative response was reversed when the IFN*β*-treated B-cells were simultaneously incubated with the STAT1 inhibitor fludarabine. These results confirm the role of IFN*β*-1b-induced STAT1 phosphorylation and SOCS1 expression in the inhibition of the antigen presenting capacity of B-cells. 

In a recent study, Tanaka et al. have demonstrated that the EAE induction was reduced in mice with a T-cell-specific SOCS1 knockout [56], which differentiated into Th1-cells, while the frequency of Th17-cells was reduced. The SOCS1-deficient T-cells exhibited a sustained STAT1 activation [56]. In the absence of an SOCS1 inhibitory effect on IFN-*γ* signaling, naïve T-cells were overresponsive to IFN-*γ* [56] and preferentially differentiated into a Th1-cell phenotype. 

Not all RR MS patients respond to IFN*β* therapy, and early identification of nonresponders is important to avoid irreversible disability progression. Comabella et al. have recently demonstrated that monocytes from RR MS nonresponders have an increased baseline level of STAT1 phosphorylation and IFNAR1 expression compared to IFN*β*-responders, which are identified by the suppression of clinical disease activity. In addition, nonresponders produced increased levels of endogenous IFN*β* [61]. However, the expression of SOCS1 and SOCS3 was similar in responders and non-responders, suggesting that these negative regulators of STAT1 activation may be expressed but functionally deficient in non-responders. 

In summary, IFN*β* induces the SOCS1 and SOCS3 protein expression in APCs. The induction of SOCS1 in B-cells leads to CD40 suppression, whereas the induction of SOCS3 inhibits the gene expression of Th17-polarizing cytokines IL-1*β* and IL-23. In naïve CD45RA T-cells, IFN*β* also induces SOCS3 which inhibits STAT3 cell signaling and a subsequent expression of the Th17-cell master regulatory transcription factor RORc.

## 6. Simvastatin-Mediated Upregulation of SOCS3

Statins are selective inhibitors of 3-hydroxy-3-methylglutaryl (HMG)-CoA reductase, an enzyme involved in the conversion of HMG-CoA to mevalonic acid. Statins have been widely used as cholesterol-lowering agents in the treatment of cardiovascular diseases. More recently, they have been found to have anti-inflammatory and immunomodulatory properties, since they inhibit DCs' maturation and antigen presentation [62, 63]. The anti-inflammatory benefits of statins are related to their ability to reduce mevalonate and the mevalonate-derived isoprenoids farnesyl pyrophosphate (FPP) and geranylgeranyl pyrophosphate (GGPP). FPP and GGPP are involved in the posttranslational modification of small G-proteins. Simvastatin may have a therapeutic potential in RR MS [64], since it has been demonstrated to decrease new CNS lesion formation by 42% after six months of treatment in comparison to the baseline magnetic resonance imaging studies [65]. Statins' immunotherapeutic mechanisms of action are currently not fully understood.

We have also reported that naïve CD45RA^+^ T-cells cultured with SNs from simvastatin-treated monocytes decreased Th17-cell differentiation. The simvastatin treatment of monocytes and DCs from RR MS patients and HC induced decreased expression of the Th17-promoting cytokines IL-1*β* and IL-23 [66, 67]. We have also identified an increase in the SOCS3 expression in simvastatin-treated monocytes [68, 69]. Statin-induced SOCS3 downregulates the expression of IL-1*β* and IL-23, creating a cytokine milieu that inhibits the Th17-cell differentiation, as shown in Figure 1. 

Previous EAE studies have indicated that statins shifted Th1 cytokine (IFN-*γ*, IL-12, and TNF-*α*) to Th2 cytokine production (IL-4, IL-5, and IL-10) [68]. A more recent study has suggested that statins inhibit human Th17-cells' differentiation and the production of the Th17 cytokines IL-17A, IL-17F, IL-21, and IL-22 [67]. Huang et al. have demonstrated that statins block STAT1 phosphorylation, which is crucial in IFN-*γ* signaling and Th1-cell differentiation [16]. SOCS3, a negative regulator of STAT1 and STAT3 activation, is increased in the statin-treated cells [66]. STAT1 phosphorylation is required for Th1 and STAT3 activation for Th17-cell differentiation. Statin-induced SOCS3 expression is proposed to downregulate the STAT1 and STAT3 phosphorylation during Th1 and Th17-cell differentiation, through which its therapeutic effect is mediated in RR MS.

## 7. Conclusions

IFN*β* and statins have been shown to be an effective treatment of RR MS. Despite different mechanisms of action, both therapies target similar signaling pathways. IFN*β* suppresses the differentiation of pathogenic Th17-cells, through its effect on the cells of the innate system (macrophages, monocytes, DCs, and B-cells), by the inhibition of the Th17-cell-promoting cytokines IL-1*β* and IL-23 via induction of SOCS3. In B-cells, IFN*β* downregulates CD40 costimulatory molecule expression through the induction of SOCS1. IFN*β* also inhibits Th17-cell differentiation directly through the suppression of RORc. 

Statins lead to SOCS3 and SOCS7 expression in the innate immune response cells. In monocytes and DCs, SOCS3 inhibits IL-1*β* and IL-23 expression, which leads to an inhibitory cytokine milieu for the Th17-cells' differentiation. Statins and IFN*β* both induce the SOCS3 expression, which is crucial in the suppression of Th17-cell differentiation and consequently for their therapeutic effect in RR MS. 

The available results have identified SOCS proteins as an attractive therapeutic target in autoimmune diseases. The designer SOCS-mimetic drugs have already been tested in an animal model of the CNS inflammatory disease. Tyrosine kinase inhibitor peptide (Tkip), a short peptide SOCS1 mimetic, both prevented and treated active EAE [69], thus representing a promising therapeutic approach that will likely be further pursued in clinical testing.
